# Regeneration of Intrabony Defects Using a Novel Magnesium Membrane

**DOI:** 10.3390/medicina59112018

**Published:** 2023-11-16

**Authors:** David Botond Hangyasi, Győző Körtvélyessy, Marko Blašković, Patrick Rider, Svenja Rogge, Stjepan Siber, Željka Perić Kačarević, Marija Čandrlić

**Affiliations:** 1 Department of Periodontology, Faculty of Dentistry, University of Szeged, Tisza Lajos krt. 64-66, H-6720 Szeged, Hungary; hangyasidavidbotond@gmail.com; 2 Department of Oral Biology and Experimental Dental Research, Faculty of Dentistry, University of Szeged, Tisza Lajos krt. 64-66, H-6720 Szeged, Hungary; kortvelyessygy@gmail.com; 3 Department of Oral Surgery, Faculty of Dental Medicine Rijeka, University of Rijeka, Krešimirova 40/42, 51 000 Rijeka, Croatia; marko_blaskovic@yahoo.com; 4 Botiss Biomaterials GmbH, 15806 Zossen, Germany; patrick.rider@botiss.com (P.R.); svenja.rogge@botiss.com (S.R.); 5 Department of Dental Medicine, Faculty of Dental Medicine and Health Osijek, J.J. Strossmayer University of Osijek, Crkvena 21, 31 000 Osijek, Croatia; siberstjepan@yahoo.com; 6 Department of Anatomy, Histology, Embryology, Pathologic Anatomy and Pathologic Histology, J.J. Strossmayer University of Osijek, Crkvena 21, 31 000 Osijek, Croatia

**Keywords:** intrabony defects, NOVAMag membrane, resorbable metal, magnesium

## Abstract

*Background and Objectives*: Due to their specific morphology, the regeneration of intrabony defects (IBDs) represents one of the greatest challenges for clinicians. Based on the specific properties of a magnesium membrane, a new approach for the surgical treatment of IBD was developed. The surgical procedure was described using a series of three cases. *Materials and Methods*: The patients were healthy individuals suffering from a severe form of periodontitis associated with IBD. Based on radiographic examination, the patients had interproximal bone loss of at least 4 mm. Due to its good mechanical properties, it was easy to cut and shape the magnesium membrane into three different shapes to treat the specific morphology of each IBD. In accordance with the principles of guided bone regeneration, a bovine xenograft was used to fill the IBD in all cases. *Results*: After a healing period of 4 to 6 months, successful bone regeneration was confirmed using radiological analysis. The periodontal probing depth (PPD) after healing showed a reduction of 1.66 ± 0.29 mm. *Conclusions*: Overall, the use of the different shapes of the magnesium membrane in the treatment of IBD resulted in a satisfactory functional and esthetic outcome.

## 1. Introduction

Intrabony defects (IBDs) are vertical defects caused by the invasion of bacterial plaque during periodontitis and the resorption of alveolar bone. Left untreated, IBDs risk further disease progression, bone loss, increased mobility of teeth and eventual tooth loss [1]. IBDs have a specific morphology, whereby the periodontal pocket forms apical to the alveolar crest next to the tooth root and is surrounded by bony walls on 1–3 sides, and they can be self-contained and non-self-contained [2].

Treatment measures for IBDs include surgical and non-surgical approaches, with each approach aiming to increase tissue volume via reconstruction or regeneration. One treatment approach uses autogenous osseous and connective tissue grafts [3]. The technique uses a split-thickness flap created from a horizontal incision from the mucogingival junction to the alveolar mucosa and then apically to the labial vestibular fold. An osseous graft is harvested locally and secured with a titanium screw and packed with cancellous bone. A connective tissue graft is retrieved from the palate and used to cover the augmentation. The main disadvantage of this surgical treatment is that it is very invasive [3,4,5]. Conventional flap design and soft tissue maturation during healing can lead to a retraction of the marginal tissues. To reduce invasiveness, minimally invasive techniques have been created to preserve the soft tissue [6].

Minimally invasive treatment techniques are used to maintain the integrity of the papilla when accessing the IBD site during surgery, minimize trauma to the periodontal tissue, and stabilize blood clots [7]. Preserving the papilla is important as their reconstruction is very challenging. The papilla only has a minor blood supply, leading to unpredictable surgical results [8]. Consequently, the integrity of the papilla is usually preserved during dental procedures to minimize its disappearance.

Surgical treatments can involve the application of biomaterials to augment the bone using barrier membranes, bone grafts, and possibly the application of biologics [9]. In a systemic review by Needleman et al., guided tissue regeneration (GTR) using only a barrier membrane showed advantages over conventional open-flap debridement in terms of attachment level change, gingival recession, and reduction in probing depth [10]. Also, it has previously been reported that the use of a barrier membrane in combination with bone graft improves the bone fill volume in the treatment of IBD [11,12]. However, complete rebuilding of the osseous architecture can be difficult to achieve [7]. This could be related to compression caused by the overlying soft tissue, restricting the growth of bone inside of the defect [13]. Nevertheless, the survival rate of teeth with IBD 10 years after GTR is 96%, and therefore it is an important procedure in the management of IBD [14].

The recent development of a pure magnesium membrane provides a new treatment possibility for IBD. The magnesium membrane has many beneficial characteristics, such as mechanical stability, a malleable structure, and a surface that enables the adhesion and migration of human gingival fibroblasts, and is completely resorbable. These properties have already been taken advantage of for establishing new techniques that were previously impossible for a resorbable membrane, such as with the magnesium membrane shield technique, recently established by Elad et al. [15].

It has previously been reported that the magnesium membrane offers good soft tissue adhesion as well as excellent mechanical stability, which, when applied to intrabony defects, offers the possibility for greater stability in rebuilding the osseous architecture as well as preserving the overlying soft tissue and papilla [16,17,18]. It has been shown in in vitro and in vivo tests that fibroblastic cells will adhere to and migrate over the surface of degrading magnesium membranes [16]. In the same study, immunohistological analysis of the cellular structure revealed that the presence of magnesium ions at concentrations of 75 mM or higher increased the focal adhesion of the cells.

Furthermore, the mechanical stability of the membrane could provide additional stability to aid with the regeneration of vertical defects. Previous benchtop testing has demonstrated that the magnesium membrane has low elasticity and high tensile strength, 183.0 ± 10.7 MPa [17]. This is substantially higher than that of other resorbable options, such as collagen (between 4.8 and 22.5 MPa [19,20]) or polymeric membranes (~14.5 MPa [21]). The ability of the membrane to maintain its shape and structure to promote bony ingrowth has been demonstrated in a guided bone regeneration model in vivo [17,18]. Used for guided bone regeneration in a Beagle dog model, the magnesium membrane secluded the defect site from an invasion of the overlying soft tissues just as efficiently as a collagen membrane used as a control [17,18]. Therefore, the membrane could be ideal for preserving elevation of the soft tissue during the critical healing period and maintaining stability at the surgical site to enable optimal bone volume. 

Despite its mechanical properties, the magnesium membrane is completely degradable [15,17,18]. As the magnesium metal degrades, it is transformed into magnesium salts and hydrogen gas, both of which are resorbed by the body [22,23]. The degradation process causes an influx of magnesium ions, which are naturally present within almost every cell of the body and are involved in many important processes [24,25]. The daily recommended intake of magnesium is between 300 and 350 mg for adults [26], which is higher than the weight of the largest pure magnesium membrane, which is approximately 280 mg [17]. Considering that the membrane is absorbed gradually over a period of months, the level of magnesium ion influx per day is relatively low. 

Based on the above properties of the magnesium membrane, the idea for a new surgical approach in the treatment of IBD was developed. The characteristics of the membrane could provide a surgical approach that is easier to perform, causes less trauma to the tissue, and is less time consuming, thereby respecting the health, function, and esthetics of the oral tissues for an improved regenerative outcome. Therefore, the aim of this paper was to demonstrate a new approach in the treatment of IBD by using a magnesium membrane. The magnesium membrane can be cut into varying shapes and designs, which demonstrates the versatility of the membrane to support the needs and requirements for the treatment of individual defects. The successful application of the membrane is reported in a case series of three patients. 

## 2. Materials and Methods

### 2.1. Material

The magnesium membrane (NOVAMag^®^ membrane, botiss biomaterials GmbH, Zossen, Germany) used in this report is made from pure magnesium, has a thickness of 140 µm, and is provided sterile. 

### 2.2. Surgical Protocol

In accordance with the Declaration of Helsinki, the Ethics Committee of the Faculty of Dental Medicine and Health Osijek granted ethical approval (Class: 602-01/23-12/05; No. 2158/97-97-10-23-03). All patients agreed to participate in the experimental surgical procedure and signed an informed consent form. 

In this article, a new surgical approach for the treatment of IBD is described, utilizing a novel resorbable magnesium membrane in three cases. The described cases provide a solution to securely stabilize the vertical augmentation of graft material as well as support the soft tissue, preventing the compression of the graft. Participants in this case series were randomly selected from the patient population of a practice specializing in periodontology. Participants were healthy, non-smoking adults between the ages of 18 and 70 years who had no contraindications to periodontal surgical treatment. Based on radiographic examination, patients were required to have interproximal bone loss of at least 4 mm to participate in the experimental procedure. All patients gave their written consent to participate in the experimental procedure and to publish the results. They were treated by the same experienced surgeon. Three months before the scheduled procedure, patients received periodontal treatment to remove supra- and subgingival plaque and calculus and were given oral hygiene instructions. Immediately prior to surgery, all participants had a full-mouth plaque score (FMPS) and a full-mouth bleeding score (FMBS) of less than 20%.

The surgical procedure began with the administration of local anesthesia (Ubistesin^®^ forte, 3M ESPE, Seefeld, Germany) and a mouth rinse with chlorhexidine gluconate (CURAPROX^®^ Perio Plus Protect CHX 0.12, Kriens, Switzerland). Regarding the interdental space width (ISW), the defect site was exposed with a simplified papilla preservation flap (ISW ≤ 2 mm), and in case 3, due to a complex intrabony defect affecting all anterior teeth, vertical incisions and a full-thickness flap were created to allow better visibility during instrumentation [27]. The intrasulcular incision was made from the base of the pocket to the crest of the bone to expose the defect site. When the defect site was exposed, granulation tissue was removed, and detailed scaling and root planing was performed using the Gracy^®^ curette set (Hu-Friedy^®^, Chicago, IL, USA) and ultrasonic scaler (Piezon^®^ Master 400, EMS, Nyon, Switzerland). 

After the IBD site has been prepared, the magnesium membrane was cut to shape with the NOVAMag^®^ scissors (Carl Martin GmbH, Solingen, Germany) so that it would bridge above the interdental defect and elevate the papilla. Cutting was also performed to ensure that the edges of the membrane were rounded and that there were no sharp points that could damage the soft tissue. The NOVAMag^®^ sculptor (Carl Martin GmbH, Solingen, Germany) was used to flatten the edge of the membrane and to bend the membrane to the contours of the IBD and the interdental space. The IBD was filled using a bovine xenograft (cerabone^®^, botiss biomaterials GmbH, Zossen, Germany), after which the magnesium membrane was positioned above the bone substitute, covering the defect and overlapping on the native oral and vestibular bone surface. The flap was then completely closed with mattress sutures and single sutures using 6-0 nylon (SMI^®^, St. Vith, Belgium, Surgicryl Rapid 6.0.). 

Antibiotics (Klavocin^®^ BID, Pliva, Zagreb, Croatia; 825 mg amoxicillin + 125 mg clavulanic acid) were prescribed to the patients to take every 12 h for seven days postoperatively. Patients were instructed to practice proper oral hygiene with additional support from 0.12% chlorhexidine gluconate twice a day for two weeks. Oral analgesics (ibuprofen à 400 mg) were prescribed for pain management. Sutures were removed 10 days after surgery, and regular monthly follow-up visits were scheduled at 30, 60, and 90 days after surgery. Between 4 and 6 months post-op, a control radiograph was obtained to evaluate the results of surgical treatment.

## 3. Results

In the next subsections, the three reported cases are described in more detail. The patients were two women and one man between the ages of 35 and 70 years. The average periodontal probing depth (PPD) was measured to be 7.5 ± 1.04 mm at baseline. The magnesium membranes proved to be very easy to handle, and no tearing occurred during shaping and insertion. After surgery, patients reported no serious complications, only mild edema at the surgical site and pain, which was successfully controlled with oral analgesics. After four to six months of healing, radiographic examination showed satisfactory bone regeneration in all three cases presented. After healing, PPD was measured to be 5.83 ± 0.75 mm, meaning that the total reduction in PPD was 1.66 ± 0.29 mm.

### 3.1. Case 1

**Figure 1:**
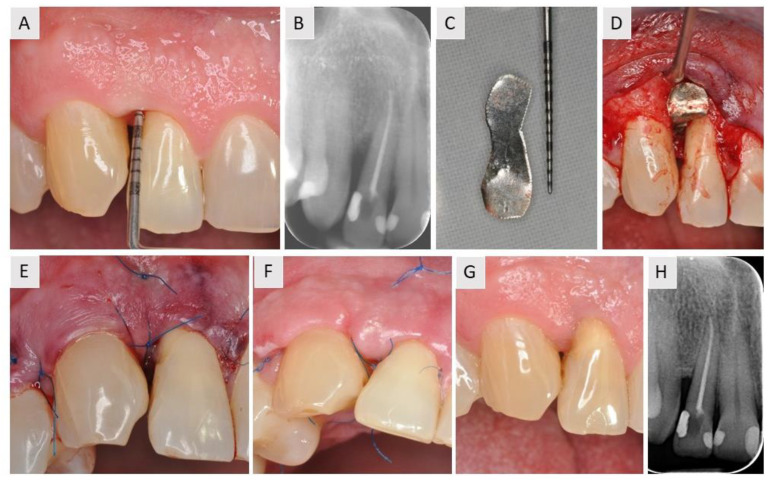
Case report of a patient who presented with a combined vertical and horizontal bone defect on the distal surface of tooth 12 (FDI Dental Numbering System for adults). (**A**) Baseline clinical situation. A probing depth of 9 mm was measured with the UNC probe. There was a gingival black triangle (GBT) in the interdental space between teeth 22 and 23. (**B**) Initial radiographic examination. Combined vertical and horizontal bone loss distal to the root of tooth 22 was noted. (**C**) After debridement, a magnesium membrane was shaped with special instruments to match the shape of the IBD. (**D**) The defect was filled with bovine xenograft and covered with magnesium membrane to fill the interdental space and elevate the interdental papilla. (**E**) The flap was adapted and sutured. (**F**) Clinical situation 10 days postoperatively. (**G**) Clinical situation after 6 months of healing. Note the healthy appearance of the interdental tissue and the minimization of the GBT between teeth 22 and 23. (**H**) Radiological examination 4 months postoperatively. Note the bone formation on the distal surface of tooth 22.

The patient was a 53-year-old man with no history of complaints regarding general health. The patient presented with combined horizontal and vertical bone loss around tooth 22 (FDI Dental Numbering System for adults), which had previously been treated endodontically. The defect was measured to be 9 mm with the UNC^®^ probe (Figure 1A). The retroalveolar radiograph confirmed the same findings as the clinical examination (Figure 1B). The magnesium membrane was cut into a rounded strip that could easily be positioned between the teeth (Figure 1C). The defect was filled with bovine xenograft and covered with a magnesium membrane that was positioned to elevate the papilla (Figure 1D). Six months after the surgical procedure, the patient showed satisfactory bone regeneration, which was confirmed using radiological examination (Figure 1H).

### 3.2. Case 2

**Figure 2:**
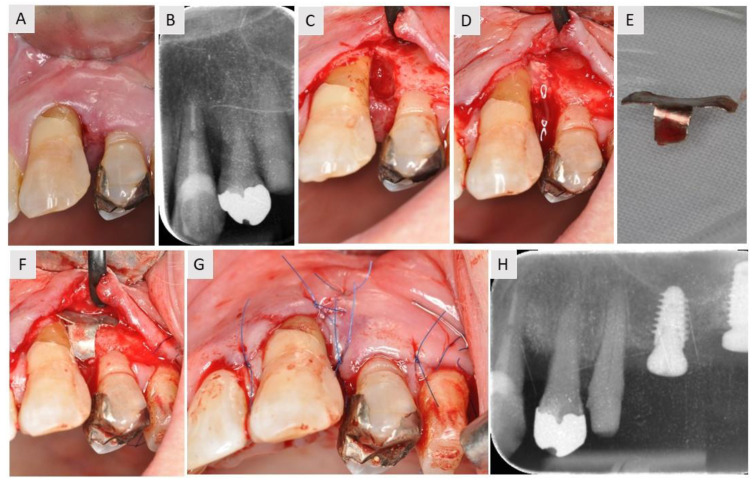
Case report of a patient who presented with an IBD between the distal root surface of tooth 23 and the mesial root surface of tooth 24. (**A**) Initial clinical situation. There was bone loss and associated loss of interdental soft tissue between teeth 23 and 24. (**B**) Initial radiographic examination. Combined vertical and horizontal bone loss was noted. (**C**) Flap elevation confirmed the radiographic findings. (**D**) After debridement, bovine xenograft was used to fill the IBD. (**E**) The magnesium membrane was cut in a T-shape to match the shape of the defect. (**F**) The defect was covered with the membrane. Note how the vertical line of the T-shaped membrane fitted into the interproximal space where the pre-existing papilla was located. (**G**) The flap was repositioned and sutured with single sutures. (**H**) Radiological examination after 6 months of healing. The radiological examination revealed satisfactory bone gain.

The patient in this case report was a 70-year-old woman with no prior history of health problems. The patient had a combined vertical and horizontal IBD in the area between teeth 23 and 24. The loss of interdental tissue was easily detected clinically (Figure 2A) and radiologically (Figure 2B). After flap elevation and debridement, the defect was filled with bovine xenograft and covered with a magnesium membrane that was T-shaped to fit the defect (Figure 2E). The wide base of the membrane enabled it to be secured to the bone, stabilizing the vertical augmentation (Figure 2F). The flap was then sutured with single sutures (Figure 2G). After six months of healing, a radiological examination was performed (Figure 2H).

### 3.3. Case 3

**Figure 3:**
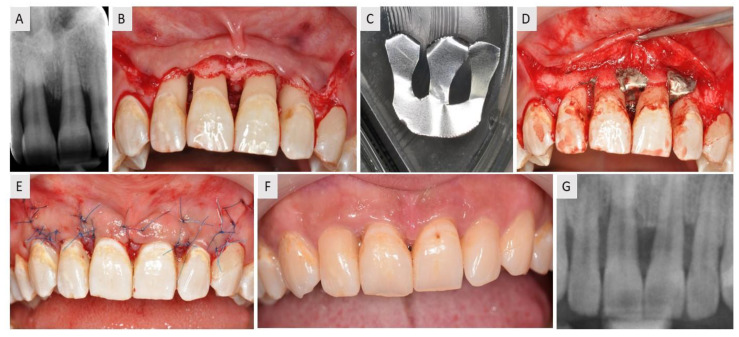
A case report of a 35-year-old female with combined vertical and horizontal defect in the esthetical zone of the maxilla. (**A**) The radiographic examination revealed severe bone loss. (**B**) Sulcular and vertical incisions were performed to elevate the flap, and the defect site was exposed. (**C**) The magnesium membrane was cut into an M-shape to cover interdental spaces between the teeth. (**D**) The defect was filled with bovine xenograft and covered with magnesium membrane that was previously shaped according to the defect. (**E**) The flap was repositioned and sutured using monofilament sutures. (**F**) Postoperative check-up after 6 months of healing. Note the healthy appearance of the soft tissues around the teeth and minimization of GBT. (**G**) Final radiological examination six months postoperatively revealed satisfactory bone healing.

In this case report, the patient was a 35-year-old female with no prior history of medical problems. The patient presented with combined vertical and horizontal bone loss in the esthetic region of the maxilla (Figure 3A). After the flap elevation and debridement (Figure 3B), the membrane was cut into an “M” shape that enabled three IBDs to be treated (Figure 3C). The “M” shape provided support to the three overlying prongs of the membrane that were used to vertically elevate the soft tissue and support the augmentation material. The defect was filled with bovine xenograft and individual prongs of the membrane were positioned interproximally between each tooth (Figure 3D). The flap was sutured using single sutures (Figure 3E). Radiological examination was undertaken after four months of healing and revealed satisfactory bone gain in the interproximal space (Figure 3G).

## 4. Discussion

Regenerative dentistry encompasses not only functional restoration, such as the rebuilding of bone prior to implant placement, but also esthetic approaches. Surgical techniques aimed at treating IBD can include bone augmentation whilst also focusing on preserving or rebuilding the soft tissue. These techniques are very technically demanding and time-consuming procedures that can have unpredictable outcomes. This means that there is yet to be a defined a procedure that can be classed as the gold standard. This paper has presented the first clinical cases where a magnesium membrane has been applied for periodontal treatment of IBD.

A common treatment modality for IBD that also focuses on rebuilding soft tissues uses autologous grafts in a very invasive procedure [8]. The development of magnesium membranes has provided the possibility for alternative treatment approaches that are less invasive and less technically demanding. The use of a magnesium membrane stabilizes the interdental bone augmentation and rebuilds the height of the soft tissue. While in alternative procedures, a soft tissue graft has to be harvested from an additional site, the magnesium membrane is synthetic, thereby reducing trauma for the patient. Additionally, as the membrane can be easily shaped using the appropriate instruments, the procedure is less time- and labor-intensive compared to the currently established techniques. These combined outcomes produce a technique that is easier and faster to perform, is less technically demanding, and is therefore more accessible. 

Looking back at the development of biomaterials and techniques in GTR, initially, non-absorbable membranes such as polytetrafluoroethylene (PTFE) or expanded polytetrafluoroethylene (ePTFE) membranes were used [28]. The advantages of these membranes were biocompatibility and good space maintenance at the augmentation site. However, a second surgical procedure was required to remove the membrane. Therefore, a second generation of membranes was developed to be resorbable. Of the resorbable membranes, the most commonly used variety are made from collagen. Collagen membranes have several advantages over non-resorbable membranes. Firstly, a second surgical procedure is avoided due their complete resorption and integration with the soft tissue [29]. In addition, these membranes have lower immunogenicity and a lower dehiscence rate [28,30]. Nevertheless, in one study, these membranes showed unpredictable resorption, which could affect bone healing [13]. Another problem with collagen membranes is that they can be easily torn by the oral surgeon during insertion or as a result of further loading conditions [19]. 

In contrast to the collagen membranes, an important property of the magnesium membrane is that it can be easily cut and adapted to the shape of the defect without the risk of tearing during placement, which was confirmed in the three cases presented here. Although made from a degradable metal that has an initially smooth shiny surface, in vitro and in vivo studies have demonstrated the ability of gingival cells to have a good adhesion to its surface [16,31]. Under physiological conditions, the degradation of magnesium releases magnesium ions, hydroxide ions, and molecular hydrogen. The presence of magnesium ions that are released during the degradation and resorption process can improve the focal adhesion of fibroblastic cells [16]. Additionally, the release of magnesium ions can also be beneficial for the migration of fibroblasts, which can quickly repopulate the surface of the degrading membrane [32]. This outcome is reproduced clinically, as it can be observed in all three of the presented cases that there was good soft tissue healing. 

Previous studies have reported the presence of gas cavities around magnesium implants as they degrade [18,33,34,35]. However, this was not replicated in this study, possibly due to the location of the membrane enabling the gas to escape via the sulcus. In all cases, the flap remained closed during the healing period and interdental soft tissues obtained a healthy appearance.

The use of bovine xenograft has been demonstrated to be a suitable alternative to the use of autologous bone for IBDs, providing similar regenerative outcomes [36], and its use is not restricted by the availability of intra-oral donor bone. It can be used in combination with resorbable membranes for IBDs, which has been described by several authors. This combination of biomaterials resulted in a higher clinical attachment level (CAL) compared with flap surgery alone. In all cases, the combination of bovine xenograft and collagen membrane resulted in an increase of at least 3 mm CAL [37]. The same group of authors also published a five-year follow-up study on the success of IBD treatment with bovine xenografts and collagen membranes. The conclusion stated that the combination of bovine xenograft and collagen membrane achieved significantly higher CAL gain over a five-year period compared to treatment with open-flap debridement alone [38]. 

However, some authors have reported that the use of a collagen membrane in combination with xenograft provides no additional benefit when applied to large vertical defects [12,39]. Nevertheless, the use of a resorbable membrane provides a better clinical outcome in comparison to open-flap debridement performed alone [12,40,41]. The lack of clinical benefit of using a collagen membrane when used in combination with a bone graft for the vertical augmentation of IBDs could be associated with its low mechanical strength and inability to resist the pressure exerted on the augmentation material from the overlying soft tissue [13].

In a recent review by Jepsen et al., it was stated that the defect morphology plays an important role in the outcome of the regenerative treatment and that the choice of biomaterial should be taken into consideration as a part of the strategy for the treatment [42]. One example provided was the use of titanium-reinforced barrier membranes to treat “non-contained” IBDs. In this instance, the use of a magnesium membrane, which is also self-supporting, could be used as an alternative without the requirement to extract the membrane later. In the review, the many other factors that need to be taken into consideration to ensure a successful outcome were stressed, such as infection control, tooth mobility, endodontic status, flap design, and appropriate postoperative care and maintenance. 

Due to the mechanical properties of the magnesium membrane, it is possible to treat individual or multiple interdental defects at once. As presented in case 1, the membrane can be cut into individual sections that are positioned over the crest of the IBDs. A singular membrane kept in one piece can treat multiple positions and can be secured to the bone for greater stability of larger defects, as demonstrated by cases 2 and 3. By keeping the membrane in one piece, it provides added stability and speed for treating multiple defects at once. However, this method requires a large flap to be used. Alternatively, if a less invasive approach is desired and the size of incision used for the flap is kept to a minimum, the individually cut sections of the magnesium membrane can be placed in single or multiple interdental spaces, as was demonstrated for case 1. 

The application of a magnesium membrane to treat IBDs is presented as a viable alternative to existing techniques. As the membrane is synthetic, it is not necessary to retrieve an autologous graft to add volume to the treatment area. The membrane is easily cut to shape and positioned in the interdental spaces, offering multiple possibilities regarding its shape and application, which can be used to treat singular or multiple IBDs. In conclusion, the use of a novel magnesium membrane cut into three different shapes has been used for the treatment of IBD, which has resulted in a reduction in PPD and therefore a satisfactory functional and esthetic outcome.

## Data Availability

The data presented in this article are available on request from the corresponding authors.

## References

[1] Shukla S., Chug A., Mahesh L., Singh S., Singh K. (2019). Optimal management of intrabony defects: Current insights. Clin. Cosmet. Investig. Dent..

[2] Hasegawa Y., Takayama T., Iwano Y. (2021). Clinical classification of tooth position in the alveolar bone housing with periodontal defects. J. Dent. Sci..

[3] Sultan N., Jafri Z., Sawai M., Bhardwaj A. (2020). Minimally invasive periodontal therapy. J. Oral Biol. Craniofacial Res..

[4] Muthukumar S., Ajit P., Sundararajan S., Rao S.R. (2016). Reconstruction of interdental papilla using autogenous bone and connective tissue grafts. J. Indian. Soc. Periodontol..

[5] Azzi R., Takei H., Etienne D., Carranza F.A. (2001). Root coverage and papilla reconstruction using autogenous osseous and connective tissue grafts. Int. J. Periodontics Restor. Dent..

[6] Ziahosseini P., Hussain F., Millar B.J. (2014). Management of gingival black triangles. Br. Dent. J..

[7] Anoixiadou S., Parashis A., Vouros I. (2023). Minimally Invasive Non-Surgical Technique in the Treatment of Intrabony Defects—A Narrative Review. Dent. J..

[8] Singh V.P., Uppoor A.S., Nayak D.G., Shah D. (2013). Black triangle dilemma and its management in esthetic dentistry. Dent. Res. J..

[9] Prato Pini P.G., Roberto R., Pierpaolo C., Carlo T., Robert A. (2004). Interdental papilla management: A review and classification of the therapeutic approaches. Int. J. Periodontics Restor. Dent..

[10] Needleman I., Worthington H.V., Giedrys-Leeper E., Tucker R. (2006). Guided tissue regeneration for periodontal infra-bony defects. Cochrane Database Syst. Rev..

[11] Kubota T., Nemoto Y., Nohno K., Nezu A., Morozumi T., Yoshie H. (2018). A Comparable Study of Combinational Regenerative Therapies Comprising Enamel Matrix Derivative plus Deproteinized Bovine Bone Mineral with or without Collagen Membrane in Periodontitis Patients with Intrabony Defects. Open J. Stomatol..

[12] Bunyaratavej P., Wang H.-L. (2001). Collagen Membranes: A Review. J. Periodontol..

[13] Elgali I., Omar O., Dahlin C., Thomsen P. (2017). Guided bone regeneration: Materials and biological mechanisms revisited. Eur. J. Oral Sci..

[14] Cortellini P., Tonetti M.S. (2004). Long-term tooth survival following regenerative treatment of intrabony defects. J. Periodontol..

[15] Elad A., Rider P., Rogge S., Witte F., Tadić D., Kačarević Ž.P., Steigmann L. (2023). Application of Biodegradable Magnesium Membrane Shield Technique for Immediate Dentoalveolar Bone Regeneration. Biomedicines.

[16] Amberg R., Elad A., Rothamel D., Fienitz T., Szakacs G., Heilmann S., Witte F. (2018). Design of a migration assay for human gingival fibroblasts on biodegradable magnesium surfaces. Acta Biomater..

[17] Rider P., Kačarević Ž.P., Elad A., Tadic D., Rothamel D., Sauer G., Bornert F., Windisch P., Hangyási D.B., Molnar B. (2022). Biodegradable magnesium barrier membrane used for guided bone regeneration in dental surgery. Bioact. Mater..

[18] Rider P., Kačarević Ž.P., Elad A., Rothamel D., Sauer G., Bornert F., Windisch P., Hangyási D., Molnar B., Hesse B. (2022). Analysis of a Pure Magnesium Membrane Degradation Process and Its Functionality When Used in a Guided Bone Regeneration Model in Beagle Dogs. Materials.

[19] Ortolani E., Quadrini F., Bellisario D., Santo L., Polimeni A., Santarsiero A. (2015). Mechanical qualification of collagen membranes used in dentistry. Ann. Ist. Super. Sanita.

[20] Coïc M., Placet V., Jacquet E., Meyer C. (2010). Propriétés mécaniques des membranes de collagne. Rev. Stomatol. Chir. Maxillofac..

[21] Milella E., Ramires P.A., Brescia E., La Sala G., Di Paola L., Bruno V. (2001). Physicochemical, mechanical, and biological properties of commercial membranes for GTR. J. Biomed. Mater. Res..

[22] Atrens A., Song G.L., Liu M., Shi Z., Cao F., Dargusch M.S. (2015). Review of Recent Developments in the Field of Magnesium Corrosion. Adv. Eng. Mater..

[23] Zheng Y.F., Gu X.N., Witte F. (2014). Biodegradable metals. Mater. Sci. Eng. R. Rep..

[24] Gröber U., Schmidt J., Kisters K. (2015). Magnesium in prevention and therapy. Nutrients.

[25] Jahnen-Dechent W., Ketteler M. (2012). Magnesium basics. Clin. Kidney J..

[26] EFSA NDA Panel (EFSA Panel on Dietetic Products Nutrition and Allergies) (2015). Scientific Opinion on Dietary Reference Values for magnesium. EFSA J..

[27] Levine R.A., Saleh M.H.A., Dias D.R., Ganeles J., Araújo M.G., Renouard F., Pinsky H.M., Miller P.D., Wang H.-L. (2023). Periodontal Regeneration Risk Assessment in the Treatment of Intrabony Defects. Clin. Adv. Periodontics.

[28] Eickholz P., Kim T.-S., Holle R. (1997). Guided tissue regeneration with non-resorbable and biodegradable barriers: 6 months results. J. Clin. Periodontol..

[29] Ren Y., Fan L., Alkildani S., Liu L., Emmert S., Najman S., Rimashevskiy D., Schnettler R., Jung O., Xiong X. (2022). Barrier Membranes for Guided Bone Regeneration (GBR): A Focus on Recent Advances in Collagen Membranes. Int. J. Mol. Sci..

[30] Rakhmatia Y.D., Ayukawa Y., Furuhashi A., Koyano K. (2013). Current barrier membranes: Titanium mesh and other membranes for guided bone regeneration in dental applications. J. Prosthodont. Res..

[31] Amberg R., Elad A., Beuer F., Vogt C., Bode J., Witte F. (2019). Effect of physical cues of altered extract media from biodegradable magnesium implants on human gingival fibroblasts. Acta Biomater..

[32] Wang L., Luo Q., Zhang X., Qiu J., Qian S., Liu X. (2020). Co-implantation of magnesium and zinc ions into titanium regulates the behaviors of human gingival fibroblasts. Bioact. Mater..

[33] Rider P., Kačarević Ž.P., Elad A., Rothamel D., Sauer G., Bornert F., Windisch P., Hangyási D., Molnar B., Hesse B. (2022). Biodegradation of a Magnesium Alloy Fixation Screw Used in a Guided Bone Regeneration Model in Beagle Dogs. Materials.

[34] Walker J., Shadanbaz S., Woodfield T.B.F., Staiger M.P., Dias G.J. (2014). Magnesium biomaterials for orthopedic application: A review from a biological perspective. J. Biomed. Mater. Res.-Part B Appl. Biomater..

[35] Oshibe N., Marukawa E., Yoda T., Harada H. (2019). Degradation and interaction with bone of magnesium alloy WE43 implants: A long-term follow-up in vivo rat tibia study. J. Biomater. Appl..

[36] Abolfazli N., Saleh Saber F., Lafzi A., Eskandari A., Mehrasbi S. (2008). A Clinical Comparison of Cenobone (A Decalcified Freeze-dried Bone Allograft) with Autogenous Bone Graft in the Treatment of Two- and Three-wall Intrabony Periodontal Defects: A Human Study with Six-month Reentry. J. Dent. Res. Dent. Clin. Dent. Prospects.

[37] Sculean A., Berakdar M., Chiantella G.C., Donos N., Arweiler N.B., Brecx M. (2003). Healing of intrabony defects following treatment with a bovine-derived xenograft and collagen membrane. J. Clin. Periodontol..

[38] Sculean A., Schwarz F., Chiantella G.C., Donos N., Arweiler N.B., Brecx M., Becker J. (2007). Five-year results of a prospective, randomized, controlled study evaluating treatment of intra-bony defects with a natural bone mineral and GTR. J. Clin. Periodontol..

[39] Chen C.-C., Wang H.-L., Smith F., Glickman G.N., Shyr Y., O’Neal R.B. (1995). Evaluation of a collagen membrane with and without bone grafts in treating periodontal intrabony defects. J. Periodontol..

[40] Tarakji B., Beshr K., Mossa H., Elkhatat E., Elkhatat A., Azzeghaiby S. (2015). Clinical and radiographic evaluation of periodontal intrabony defects by open flap surgery alone or in combination with Biocollagen^®^ membrane: A randomized clinical trial. J. Int. Soc. Prev. Community Dent..

[41] Lee I.K., Choi H.S., Jeong S.H., Lee J.T. (2021). Evaluating Change of Marginal Bone Height with Cone-Beam Computed Tomography Following Surgical Treatment with Guided Tissue Regeneration (Bone Grafting) or Access Flap Alone: A Retrospective Study. Medicina.

[42] Jepsen K., Sculean A., Jepsen S. (2023). Complications and treatment errors related to regenerative periodontal surgery. Periodontol. 2000.

